# Special Issue “Textile-Based Advanced Materials: Construction, Properties and Applications”

**DOI:** 10.3390/ma13245766

**Published:** 2020-12-17

**Authors:** Avinash P. Manian, Thomas Bechtold

**Affiliations:** Research Institute of Textile Chemistry/Physics, University of Innsbruck, Hoechsterstrasse 73, 6850 Dornbirn, Austria

Developments in the science and technology of textiles is not only limited to apparel and fashion. Certainly, there are research efforts on improving the construction and processing of textiles for clothing–such as on cleaner production to reduce environmental impact, increasing the utilization of fibers and process chemicals from renewable resources, and on the recycling of materials from post-consumer waste apparel back into the manufacture of new clothing articles. In addition, technological concepts developed for the creation of clothing over centuries, are now being investigated for use in a diverse array of fields—such as in the manufacture of engineering composites, personal protective equipment, and medicine. Further, developments in other fields–such as electronics, nanotechnology, and information and communication technologies–are being investigated for their incorporation into apparel and clothing to create “smart textiles”. The aim of this Special Issue was to put together a collection of scientific reports on such efforts, to highlight the range of scientific and technological questions that are being targeted, and the ingenuity of the methodologies employed to find answers. It is hoped that readers of this issue will come away with an appreciation of the research being conducted in this area, and perhaps gain inspiration for their own scientific endeavors.

The issue contains eleven research articles, on composites, geotechnical engineering, medical applications, high-performance polymers, and the development and integration of sensors in textile articles. Furthermore, there are two review articles–one on the state of technology in incorporation of nanoparticle into apparel and clothing textiles including potential safety and toxicological concerns, and the other on the integration of temperature sensors in apparel and clothing. 

Spencer et al. [1] studied microbially induced calcium carbonate precipitation (MICP) in silica sand, and what effect the incorporation of jute fibers into the sand may have on the process. MICP or bio-cementation is a process where non-pathogenic bacteria are employed to catalyze the precipitation of calcium from ground water as its carbonate salt between sand particles, so that the precipitates bind the sand and increase its strength. They found that the efficiency of calcium conversion to CaCO_3_ increased in the presence of jute, which they attributed to a possible positive influence of the fibers on fixation and viability of the microbes. The uniaxial (or unconfined) compressive strength of the jute-sand mixtures were greater than those of sand alone, but it was difficult to ascertain the contributions arising from the fibers alone compared to the greater contents of precipitated CaCO_3_. A further goal was to investigate whether the fibers would retain viable bacterial cultures and thereby exert a ‘self-healing’ effect through the facilitation of further CaCO_3_ precipitation to fill in cracks that developed over time. However, the results proved inconclusive. 

In a similar vein, Zhou et al. [2] investigated woven carbon mesh as a textile reinforcement for constructions of mortar on their uniaxial tensile behavior, and also studied the effect of dispersing 12–15 mm long copper-coated steel fibers in the mix as additional reinforcement. The tensile strength increased with the volume fraction of the textile reinforcement, but at high reinforcement levels, the cracking propensity of constructions increased, which indicated a heightened risk of delamination between the mortar and the reinforcement. Applying a pre-stress to the carbon mesh on integration into the mortar mix increased the load at which the crack first appeared in tests, but the dispersion of short length steel fibers both reduced the length and width of cracks and increased the tensile strength significantly. That suggests that the short-length fibers acted to improve reinforcement-matrix bonding and reduced the delamination risks.

Two papers in the issue deal with modeling. One is on the in-plane shear deformation behavior of textile fabric reinforcements, which if predicted with high reliability, can significantly improve the efficiency in the design and construction of composites. The aspect dealt with by Rothe et al. [3] was the critical point at which shear-induced folds occur in extension tests. In normal course, that point is estimated from inflections in load-extension profiles but that is not always valid, as visual observations have shown folds to form before an inflection occurs. The authors report on the development of a test apparatus and laser measurement system that can detect and quantify the appearance of folds in extension tests, and the measured parameters can be fed into software, also developed by the authors, to more accurately characterize the load-bearing and deformation characteristics of the reinforcements. The other is on the compression behavior of vacuum-assisted resin-impregnated composites of multi-layered woven flax fiber composites, by Hu et al. [4]. Flax shows promise as a bio-based alternative to synthetic materials as reinforcement in lightweight composites, and work such as theirs on understanding the dynamic mechanical behavior of flax-reinforced composites will contribute to an increase in confidence on their use in engineering applications.

A way to improve the biocompatibility of polyethylene terephthalate (PET) for medical applications was investigated by Montoya-Villegas et al. [5] who studied the grafting of PET fabrics with 2-hydroxyethyl methacrylate (HEMA) and co-polymer of HEMA and polyethylene glycol methacrylate under irradiation with UV or gamma-ray. The resulting substrates were then loaded with silver nanoparticles and then tested for their antimicrobial activity against gram positive and gram negative bacteria. The grafting under UV irradiation was found to yield a homogeneous, thin coating of the graft polymer whereas far thicker coatings were obtained under gamma-ray. Silver nanoparticles could be synthesized in situ on the grafted substrates from both treatments, and the resulting materials exhibited significant activity against both microorganisms. In tandem, one may be interested to read the review by Saleem et al. [6] on the application of nanomaterials in the textiles sector for end-uses ranging from improving comfort and aesthetics to UV protection and anti-static finishing. The authors also summarize the state of knowledge on the potential risks of nanomaterials to both humans and the environment.

A second investigation on application of PET fabrics in medicine is reported by Pekkanen-Mattila et al. [7], who studied their use as scaffolds for the growth of cells of the human heart muscle (cardiomyocytes). The hypothesis was that the three-dimensional structure of the woven substrates could help in the proliferation, differentiation and maturation levels of the cells. A variety of weave patterns were investigated, and all were found to support cell growth, but no one pattern appeared to present an advantage over another. The culturing of these cells on fabrics improved their structural properties, and it is hoped that the results will be beneficial in the development of materials to obtain reliable cell cultures for toxicology, drug screening and disease modeling investigations.

Ullrich et al. [8] report on investigations of conductive viscose fibers produced by the incorporation of carbon black, and their use as pressure sensors when converted to needle-punched webs together with polyester fibers. The conductivity of fibers increased with the addition of carbon black, and when these fibers were assembled into webs together with virgin polyesters, they exhibited a variation in resistance as a function of pressure with a relationship that did not change significantly over multiple pressure cycles. The color of the fibers, black, will limit their application in visible areas of apparel and additional work is required to develop means of protecting these fibers against mechanical damage and atmospheric moisture. However, these results are a source of optimism for the authors that such constructions could be employed in applications such as pressure pads inside bandages. Another investigation on imparting conductivity to textile fibers was reported by Karbownik et al. [9] who investigated the effects of incorporating polyaniline in polyacrylonitrile. Two modes were attempted: the addition of preformed polyaniline to the polyacrylonitrile spin dope with, and in situ synthesis of polyaniline in the polyacrylonitrile spin dope. The simple mixing of preformed polyaniline produced fibers exhibiting good mechanical strength, but the fibers behaved as dielectrics, and significant levels of polyaniline could not be detected. The in situ synthesis led to fibers exhibiting poor mechanical strength but high electrical conductivity, and high levels of polyaniline could be detected in the fibers. The authors recognize that further work is required to improve the process but hope that ultimately, such fibers may be used to integrate conductive lines in textiles.

Ibanez-Labiano et al. [10] report on their investigations into measurements of the dielectric properties of cotton textiles as a function of temperature, which according to the authors has not been reported previously. They employed two methods, a resonant perturbation and resonator method, and found the dielectric constant to change linearly with the temperature up to 50 °C. An innovative experimental design created for their experiments, a microstrip patch antenna, exhibits potential for use as a passive temperature sensor that can be integrated into textile structures.

Kim et al. [11] report on their work to create light emitting diode-based textile displays with polyethylene terephthalate substrates. They describe their efforts to enhance the aperture ratio, i.e., the area ratio of a light emitting diode to the total pixel area. That was achieved through a combination of reducing roughness of the substrate by depositing alternating layers of polyurethane and acryl polymers, creating a protective layer with a combination of polyvinyl alcohol (neat and derivatized), and improving conductivity through the use of carbon nanotubes.

High performance fibers often are reported for their poor dyeability that may act to limit their application, and Shao et al. [12] report on their work to improve the dyeability of polyimide fibers with the assistance of swelling agents. The addition of acetophenone, N-methyl formamide or phenoxyisopropanol to dyebaths significantly improved the dye uptake without significant evidence of volume swelling in the fibers, and the fibers exhibited good fastness to washing. 

Root et al. [13] present a review of the literature on the integration of thermocouples into textile substrates as temperature sensors, and compare methods, such as the screen printing of conductive polymers, soldering and sputter deposition of metals, the use of conductive glues and interweaving of metal wires. They also highlight the work with electroless metal deposition to achieve a conductive coating on fabric surfaces. They compare different sensing principles, such as resistance temperature detector, fiber Bragg grating, etc. Finally, they discuss the challenges that exist in such technologies, related primarily to the robustness of the integrated materials to the environment (pH, light and mechanical forces) as well as the need to maintain maximum comfort to the wearer.

The guest editors thank all authors for their valuable contributions and are grateful for the efforts they undertook to submit their contributions. Thanks also go to the reviewers who contributed with insightful and constructive comments that helped improve the overall quality of the presented work, and to the editorial staff at the publishers who ably supported the authors, reviewers and us through the whole process.
